# Improvement of Spatial and Non-verbal General Reasoning Abilities in Female Veterinary Medical Students Over the First 64 Weeks of an Integrated Curriculum

**DOI:** 10.3389/fvets.2019.00141

**Published:** 2019-05-22

**Authors:** Juan Claudio Gutierrez, Steven D. Holladay, Boaz Arzi, Christina Clarkson, Roxanne Larsen, Sakti Srivastava

**Affiliations:** ^1^School of Veterinary Medicine, University of California, Davis, Davis, CA, United States; ^2^College of Veterinary Medicine, University of Georgia, Athens, GA, United States; ^3^College of Veterinary Medicine, University of Minnesota, St. Paul, MN, United States; ^4^School of Medicine, Stanford University, Stanford, CA, United States

**Keywords:** spatial ability, anatomy, non-verbal reasoning, curriculum, visuo-spatial ability

## Abstract

Spatial visualization ability is defined as the ability to mentally rotate two- and three-dimensional figures. Visual reasoning is the ability to manipulate mental images of an object to reach a certain conclusion and has been linked to spatial ability. There is currently limited information about how entry-level spatial and visual reasoning abilities may be enhanced with progression through the rigorous veterinary medical curriculum. The present study made use of two tests that measure spatial ability and one test that measures non-verbal general reasoning ability in female veterinary students: Guay's Visualization of Views Test, Adapted Version (VVT), Mental Rotations Test (MRT), and Raven's Advanced Progressive Matrices Test, short form (APMT). Tests were given immediately before commencing the integrated veterinary medical curriculum (T0), at week 32 (T1), and at week 64 (T2) into the program. Results showed improved spatial visualization ability as measured by VVT and MRT and improved non-verbal general reasoning ability as measured by APMT at both 32 and 64 weeks. The spatial ability scores measured by VVT and MRT showed a positive correlation with non-verbal general reasoning ability scores (APMT), supporting the idea that these abilities are linked.

## Introduction

Lufler et al. (1) suggested that either the innate or learned visual–spatial ability of students may be important for mastery of the medical curriculum (1, 2). Garg et al. specifically suggested that ability to master spatial visualization might be a prerequisite for certain medical specialties, including surgery and diagnostic imaging (3). Relevant spatial visualization and visual reasoning skills have been measured by standardized tests of ability to mentally transform/rotate 3D objects in space and correctly recognize sequentially rotated objects and patterns (4–8).

Regarding spatial ability and the highly visual anatomic learning, it has been suggested that medical students either possess or acquire higher spatial ability than non-medical science students, and also show greater improvement in spatial ability scores than other science students after as little as 1 month of learning anatomy. Like all populations, medical students show differences among themselves in terms of spatial ability. It remains unknown if such differences may be innate or curriculum driven. Anatomic learning has suspected involvement because it requires the ability to visualize multiple planes (e.g., sagittal, transverse, dorsal) and the associated anatomy and to mentally visualize two-dimensional images (e.g., radiographs, CT and magnetic resonance images, ultrasound) as the 3D equivalent from which they derive (9). Regarding medical residency programs, Birchall proposed that special consideration should be given to the testing of visuospatial ability as part of the selection process for prospective applicants to radiology training programs (10).

Fernandez et al. also hypothesized that spatial ability may be enhanced through mental processes that are a required part of developing anatomic proficiency (11). These authors divided a group of clinical anatomists by anatomic skill level as experts, intermediates, novices, and unexposed controls. Spatial testing revealed that those ranked as either anatomy experts or intermediates had higher spatial visualization skills than did the anatomy novices. Further, the anatomy experts, intermediates, and novices all tested higher than controls in terms of their speed of response for making correct spatial judgments. While suggestive, these studies again did not rule out the possibility that innate spatial ability is a selective factor for developing anatomic proficiency, as opposed to or in addition to the hypothesis that spatial ability increases in direct proportion to anatomic learning.

Regarding the relationship between spatial ability and student veterinary anatomy performance, Provo et al. made use of the Purdue Visualization of Rotations Test and found a significant correlation between spatial ability scores and first-year veterinary student performance on canine anatomy examinations (12). A different report by Gutierrez et al. describes an inconclusive dispersion and inconsistency of significant positive correlation between anatomical grade and spatial and visual reasoning scores in first-year veterinary medical students, suggesting these abilities either do not correlate with grade or are overcome with progression through the anatomy courses in an integrated curriculum (13).

There are different tests that measure spatial ability and general visual reasoning. Guay's Visualization of Views Test (VVT) and the Mental Rotation Test (MRT) are two commonly used and well-validated measures of spatial ability. Regarding non-verbal general reasoning ability, a positive correlation has been observed between this ability and spatial ability in veterinary medical students at their entry-level scores, suggesting these abilities are linked (13). The Raven's Advanced Progressive Matrices Test, short form (APMT), was the test used by these authors to measure non-verbal general reasoning ability and has been similarly used by other authors (6, 7, 13–15).

In our previous study of spatial and general non-verbal reasoning ability, the female (*N* = 70), but not the male (*N* = 11), first-year veterinary students showed significant increases in both abilities (15). The males showed trends in the same direction that were non-significant, possibly a result of low sample size. The present study was designed to examine persistence of the increased test outcomes in a second cohort of first-year female students, with the males precluded due to insufficient sample size.

The hypotheses being tested in the present study were as follows: (1) there is an improvement (>1 year) (significant increase) of spatial visualization and/or general non-verbal reasoning ability in female veterinary medical students as a consequence of progression through the veterinary medical curriculum, and (2) spatial and non-verbal general reasoning abilities are long-term linked abilities.

## Materials and Methods

The present study made use of three previously validated testing tools (8) of spatial and general non-verbal reasoning. Tests were given to 43 out of 140 first-year female veterinary medical students (range of age: 20–36 years old) immediately before commencing the integrated veterinary medical curriculum (T0), at week 32 (T1), and at week 64 (T2) into the program. All 140 students were invited to participate, and 43 elected to do so. The tests required a total of 34 min to complete. The demographic characteristics of the students who participated resembled the demographic characteristics of the entire class (Hispanic/Latino: 4%, multiethnic: 15%, Asian: 20% and White/Caucasian: 61%).

The integrated veterinary medical curriculum has been designed for the adult learner to foster the development of critical thinking, problem solving, and communication skills within a comprehensive foundation of biomedical and clinical experiential learning. Veterinary anatomy instruction occurs within the first 10 blocks of the integrated curriculum (each block has an average length of 6 weeks) and is the greatest portion of the early curriculum that requires hands-on manipulation and studying of three-dimensional objects. For this reason, anatomic study has been suspected as the main factor benefiting from or perhaps increasing spatial reasoning; however, it is at the same time inseparable from the rest of the early curriculum.

The students were exposed to an average of 95 h of gross anatomy laboratories during the 64 weeks of the study. During the 64 weeks, labs were distributed across seven major blocks of the curriculum:

VET401: Basic Foundations
VET403: Musculoskeletal
VET404: Neurosciences/Senses/Behavior
VET405: Gastrointestinal and Metabolism
VET408: Cardiorespiratory
VET409: Renal
VET410: Endocrinology/Reproduction

In relation to the logistical organization of the applied anatomy laboratories at the School of Veterinary Medicine of the University of California, three students form a dissection team. Dissection teams are responsible for a canine cadaver. The learning outcomes, lab objectives, and instructional video are given to the students in an online anatomy laboratory syllabus platform. Each dissection table includes a computer screen attached to the end of the table. Each dissection team receives an iPad to access the online anatomy laboratory syllabus.

Participation of the students in this study was voluntary, and participants could withdraw from the study at any time. This study was considered as exempt from formal review by the University of California Institutional Review Board. The 43 female students who agreed to participate in the research project did so after reviewing the research protocol information. Classes of veterinary students are typically 85–93% female, with the result of insufficient male volunteers for needed statistical power.

The testing tools employed were provided in an online platform by Stanford University, Division of Clinical Anatomy. Stanford University has selected and established the use of the following tests with human medical and dentistry students.

1. Guay's Visualization of Views Test, Adapted Version (VVT): This test measures spatial ability. The test measures the ability to correctly recognize 3D objects viewed from different positions. It includes 24 questions, is timed at 8 min to complete, and is a modified and validated version of the Purdue Visualization of Views Test (www.silc.northwestern.edu). Questions present rotated images of -D objects suspended in a transparent cube. The student must identify the correct corner of the cube from which a virtual picture of the suspended object was taken. The picture of the suspended object is shown above the cube in each question. Incorrect answers incur a penalty of 1/6 of a point, making the possible range of scores from −4 to 24. Average scores reported for this test are 14.3 in fourth-year dentistry students and 10.6 in undergraduate students (16, 17).
2. Mental Rotations Test (MRT): This test also measures spatial ability. The test requires selection of three-D objects that are identical in shape to a reference object but shown in different rotational orientations (www.silc.northwestern.edu). This test therefore measures the ability to mentally rotate complex 3-D shapes in order to find a match. The 20-item test is administered in two parts timed at 3 min each, for a total of 6 min. Participants receive 1 point for each correct answer and −1 point for each incorrect answer, giving a range of possible scores from −40 to 40. Average scores described by Vandenberg and Kuse for undergraduate students are 19.06 for men and 13.17 for women (5).
3. Raven's Advanced Progressive Matrices Test, short form (APMT): This test measures non-verbal general visual reasoning ability. This 12-question, 12-min test is a subset of the original full-length Raven's Advanced Progressive Matrices Test, which was validated by Bors and Stokes. The test requires correct identification of the missing pattern in a complex design of patterns or diagrams from a set of eight choices. Students are not penalized for guessing, such that scores fall between 0 and 12. Bors and Stokes reported an overall mean score for this test of 7.39 in university students (6).

### Statistical Analysis

Descriptive statistics and a Shapiro–Wilk test were performed to assess normality of data. As data were not normally distributed, a Wilcoxon signed rank test was used to compare VVT, MRT, and APMT scores at the three times of data collection. Spearman's correlation was performed to determine if spatial ability scores correlate with non-verbal general reasoning scores at all three times of data collection. The Spearman correlation ranges from −1 to 1, with 0 indicating there is no tendency for the first variable to increase or decrease as the second variable increases. Effect size for paired comparison was determined to evaluate tendency for student scores to increase over time. Commercially available statistical software was used to perform the data analysis (JMP, Version 14, Cary, NC). For all analyses, a value of *p* < 0.05 was considered significant.

## Results

A total of 43 female students completed all three tests. Wilcoxon signed rank results revealed that mean scores on the VVT showed a significant increase between T0 and T1, from 12.3 ± 6.1 to 16.3 ± 5.5 (*p* < 0.0001, effect size *r* = 0.5), and a significant increase between T0 and T2, from 12.3 ± 6.1 to 16.9 ± 5.1 (*p* < 0.0001, effect size *r* = 0.6). Mean scores on the MRT significantly increased between T0 and T1, from 16.4 ± 7.1 to 20.2 ± 9.1 (*p* < 0.05, effect size *r* = 0.1), and between T0 and T2, from 16.4 ± 7.1 to 21.7 ± 8.4 (*p* < 0.0001, effect size *r* = 0.5). The APMT scores significantly increased between T0 and T1, from 8.0 ± 2.1 to 9.0 ± 1.5 (*p* < 0.0001, effect size *r* = 0.5), and significantly increased between T0 and T2, from 8.0 ± 2.1 to 9.1 ± 2.2 (*p* < 0.0001, effect size *r* = 0.6). Effect size for the paired comparison between T1 and T2 was *r* = 0.4, *r* = 0.1, and *r* = 0.5 for VVT, MRT, and APMT, respectively. All the tests showed a numeric (non-significant) score increase between T1 and T2. Changes in scores are summarized in Table 1.

**Table 1:** Wilcoxon signed rank test results: Mean ± SD of T0, T1, and T2 scores for the three tests from 43 female veterinary medical students.

	**VVT (Max. possible score: 24)**	**MRT (Max. possible score: 40)**	**APMT (Max. possible score: 12)**
T0	12.3 ± 6.1	16.4 ± 7.1	8 ± 2.1
T1	16.3 ± 5.5^**^	20.2 ± 9.1^*^	9 ± 1.5^**^
T2	16.9 ± 5.1^**^	21.7 ± 8.4^**^	9.1 ± 2.2^**^

* p < 0.05;

** *p < 0.0001. VVT, Visualization of Views Test, Adapted Version; MRT, Mental Rotations Test; APMT, Raven's Advanced Progressive Matrices Test, short form*.

When performing Spearman's correlation for the VVT/APMT and the MRT/APMT scores, a positive correlation between spatial and general non-verbal reasoning abilities was detected at the three times of the study (Table 2).

**Table 2:** Spearman's correlation for VVT vs. APMT and MRT vs. APMT at T0, T1, and T2 for 43 female veterinary medical students.

	**T0 (Spearman's p)**	**T1 (Spearman's p)**	**T2 (Spearman's p)**
VVT/APMT	+0.4	+0.4	+0.4
MRT/APMT	+0.4	+0.4	+0.2

## Discussion

In the present study, female veterinary students showed increased scores on the two tests of spatial ability and the one test of general non-verbal reasoning ability at week 32, and that increase was maintained until week 64 into the curriculum, suggesting progression through the rigorous veterinary curriculum persistently increases both spatial and non-verbal general reasoning ability. Test scores at week 64 were, numerically, modestly higher and *p*-values modestly lower in all cases compared to week 32, suggesting the possibility that longer time in the curriculum may further increase the measured abilities, or, alternately, these abilities may be plateauing. Effect size results suggest a tendency for the students to increase test scores over time.

It has been suggested that anatomic learning requires the need to mentally translate and rotate 3D structures. It has been also suggested that the anatomy instruction may underlie increases in spatial ability that have been detected during the early human medical college curriculum (9, 11, 18). Studies by Lufler et al. (1) support this idea, as do the results presented in the present manuscript. Luffler et al. found that medical students experienced significant visual spatial benefits during participation in the medical gross anatomy courses in the medical program (1). Similarly, dental education appears to enhance performance of spatial ability tasks that are specific to dentistry, suggesting that it leads to the development of spatial mental methods of learning anatomy (17).

Regarding correlations between spatial ability and anatomical learning, spatial ability may be a significant factor. However, it may not be as important in undergraduate contexts where the models of learning anatomy are less rigorously dependent on hands-on cadaver dissection (19). Studies by Provo et al. reveal an improvement in spatial test scores in first-year female veterinary students at 8 months into the curriculum (12). This supports the present study with similar observations at ~16 months into the DVM integrated curriculum and likely also agrees with the same increases in general non-verbal reasoning ability detected with the APMT.

When performing Spearman's correlation to understand the relationship between spatial ability scores (VVT and MRT) and non-verbal general reasoning scores (APMT), a positive correlation was found between the VVT and APMT scores and between the MRT and APMT scores at all times of the experiment. This confirms that spatial ability and non-verbal general reasoning abilities are linked in the long term. Studies by Keehner et al. support this statement. The authors reveal that university students (not involved in medical programs) with high performance in both spatial and non-verbal reasoning tests are more rapid learners of a surgical laparoscopic technique by virtual reality than those with lower performance (20).

In the present study, the improvement of non-verbal general reasoning abilities measured by APMT was detected at week 32, and it was maintained at week 64 into the veterinary medical curriculum. These results were linked to the results for spatial ability. This evidences a pattern for long-term improvement of these abilities that has to be further explored at even later times in the curriculum. Regarding the long-term improvement of reasoning abilities (measured by the ability to solve problems that follow serial patterns) in older adults, reasoning training resulted in less functional decline of instrumental activities of daily living. The cognitive training resulted in long-term improvement in cognitive abilities that continued for 5 years after the beginning of the study (21).

A possible limitation of this study is that results were obtained from only one academic institution. Similar pooling of data from first-year veterinary students at two or preferably more collaborating colleges of veterinary medicine may confirm these findings. Another potential limitation of this study is the voluntary participation of the students; participating students could have felt more confident in their spatial and visual reasoning abilities than non-participating students, possibly serving as a confounder of the results. Also, the lack of male participants is a limitation in the study considering the possible difference in gender learning preferences.

Additional studies are being performed to determine if visual and related abilities continue to improve later in the curriculum, plateau after year 1, or decrease as time passes during the veterinary integrated medical curriculum. It is therefore planned to test the present or a new cohort additionally at the middle and end of their second year in the veterinary curriculum. Future studies should also include the relationship between these abilities and the mastery of the visual-intense disciplines of diagnostic imaging and surgery during the fourth or clinical year of the curriculum.

## Conclusion

The present study concluded that (1) first-year female veterinary medical students who have progressed through 64 weeks of an integrated curriculum, which contained substantial anatomy dissection and learning experience, demonstrated improvement of their spatial visualization ability as measured by VVT and MRT; (2) these students also demonstrated improvement at week 64 in non-verbal general reasoning ability as measured by APMT; and (3) spatial ability scores measured by VVT and MRT showed a positive correlation with non-verbal general reasoning ability scores (APMT) at all times of the study, providing additional evidence that these abilities are linked in the long term.

## Ethics Statement

The study only uses online testing in veterinary students. The study was considered exempt from institutional IRB review (University of California, Davis).

## Author Contributions

JG: literature review, writing, data analysis, and final organization of the manuscript. SH: writing and data analysis. BA, CC, RL, and SS: writing.

### Conflict of Interest Statement

The authors declare that the research was conducted in the absence of any commercial or financial relationships that could be construed as a potential conflict of interest.

## References

[1] Lufler RS Zumwalt AC Romney CA Hoagland TM. Effect of visual-spatial ability on medical students' performance in a gross anatomy course. Anat Sci Educ. (2012) 5:3–9. 10.1002/ase.264 22127919

[2] Lohman D Spatial ability: a review and reanalysis of the correlational literature (technical report number 8). In: Aptitudes Research Project. Stanford, CA: School of Education; Stanford University (1979).

[3] Garg AX Norman G Sperotable L. How medical students learn spatial anatomy. Lancet. (2001) 357:363–4. 10.1016/S0140-6736(00)03649-7 11211004

[4] Elliot J Smith IM An International Dictionary of Spatial Tests. Windsor: The NFER-Nelson Publishing Company, Ltd. (1980).

[5] Vandenberg SG Kuse AR. Mental rotations, a group test of three-dimensional spatial visualization. Percept Mot Skills. (1978) 47:599–604. 724398 10.2466/pms.1978.47.2.599

[6] Bors D Stokes TL Raven's advanced progressive matrices: norms for first-year university students and the development of a short form. Educ Psychol Meas. (1998) 58:382–98.

[7] Raven J. The Raven's progressive matrices: change and stability over culture and time. Cogn Psychol. (2000) 41:1–48. 10.1006/cogp.1999.0735 10945921

[8] Hegarty M Components of spatial intelligence. In: Ross BH editor. The Psychology of Learning and Motivation. San Diego, CA: San Diego Academic Press (2010). p. 265–97.

[9] Vorstenbosch MA Klaassen TP Donders AR Kooloos JG Bolhuis SM Laan RF. Learning anatomy enhances spatial ability. Anat Sci Educ. (2013) 6:257–62. 10.1002/ase.1346 23349122

[10] Birchall D. Spatial ability in radiologists: a necessary prerequisite? Br J Radiol. (2015) 88:20140511. 10.1259/bjr.20140511 25756868 PMC4628467

[11] Fernandez R Dror IE Smith C. Spatial abilities of expert clinical anatomists: comparison of abilities between novices, intermediates, and experts in anatomy. Anat Sci Educ. (2011) 4:1–8. 10.1002/ase.196 21265030

[12] Provo J Lamar C Newby T Using a cross section to train veterinary students to visualize anatomical structures in three dimentions. J Res Sci Teach. (2002) 39:10–34. 10.1002/tea.10007

[13] Gutierrez JC Holladay SD Arzi B Gomez M Pollard R Youngblood P. Entry-level spatial and general non-verbal reasoning: can these abilities be used as a predictor for anatomy performance in veterinary medical students? Front Vet Sci. (2018) 5:226. 10.3389/fvets.2018.00226 30320127 PMC6167549

[14] Carpenter PA Just MA Shell P. What one intelligence test measures: a theoretical account of the processing in the Raven Progressive Matrices Test. Psychol Rev. (1990) 97:404–31. 2381998

[15] Gutierrez JC Chigerwe M Ilkiw JE Youngblood P Holladay SD Srivastava S. Spatial and visual reasoning: do these abilities improve in first-year veterinary medical students exposed to an integrated curriculum? J Vet Med Educ. (2017) 44:669–75. 10.3138/jvme.0915-158R3 28534720

[16] Keehner M Hegarty M Cohen C Khooshabeh P Montello DR Spatial reasoning with external visualizations: what matters is what you see, not whether you interact. Cogn Sci. (2008) 32:1099–132. 10.1080/03640210801898177 21585445

[17] Hegarty M Keehner M Khooshabeh P Montello D How spatial abilities enhance, and are enhanced by, dental education. Learn Individ Differ. (2009) 19:61–70. 10.1016/j.lindif.2008.04.006

[18] Berney S Betrancourt M Molinari G Hoyek N. How spatial abilities and dynamic visualizations interplay when learning functional anatomy with 3D anatomical models. Anat Sci Educ. (2015) 8:452–62. 10.1002/ase.1524 25689057

[19] Sweeney K Hayes JA Chiavaroli N. Does spatial ability help the learning of anatomy in a biomedical science course? Anat Sci Educ. (2014) 7:289–94. 10.1002/ase.1418 24227747

[20] Keehner M Lippa Y Montello D Tendick F Hegarty M Learning a spatial skill for surgery: how the contributions of abilities change with practice. Appl Cogn Psychol. (2006) 20:487–503. 10.1002/acp.1198

[21] Willis SL Tennstedt SL Marsiske M Ball K Elias J Koepke KM. Long-term effects of cognitive training on everyday functional outcomes in older adults. JAMA. (2006) 296:2805–14. 10.1001/jama.296.23.2805 17179457 PMC2910591

